# Low-Grade Myofibroblastic Sarcoma of the Oral Cavity: A Report of Three Cases Illustrating an Emerging Disease in Children

**DOI:** 10.3390/dermatopathology8010001

**Published:** 2021-01-01

**Authors:** Primali Rukmal Jayasooriya, Chamara Athukorala, Manjula Attygalla, Balapuwaduge Ranjit Rigobert Nihal Mendis, Tommaso Lombardi

**Affiliations:** 1 Department of Oral Pathology, Faculty of Dental Sciences, University of Peradeniya, Peradeniya 20400, Sri Lanka; primalij@yahoo.com; 2 Oral and Maxillofacial Surgery Unit, Base Hospital, Badulla 9000, Sri Lanka; chamsatukorala@yahoo.com; 3 Department of Oral and Maxillofacial Surgery, Faculty of Dental Sciences, University of Peradeniya, Peradeniya 20400, Sri Lanka; manjuatty@yahoo.com; 4 Laboratory of Oral and Maxillofacial Pathology, Unit of Oral Medicine & Oral Maxillofacial Pathology, University Hospitals of Geneva and Faculty of Medicine, University of Geneva, 1211 GE 4 Geneva, Switzerland; ranjitm@bluewin.ch

**Keywords:** low-grade myofibroblastic sarcoma, oral cavity, mandible, gingiva, malignancy, immunohistochemistry

## Abstract

Low-grade myofibroblastic sarcoma (LGMS) is a mesenchymal tumor of myofibroblasts that occurs more frequently in adults. A series of three cases is presented to illustrate that LGMS may also occur within the oral cavity in children and adolescents. The first case (Case 1) occurred intra-osseously in the mandible, while the remaining two presented as gingival swellings and were purely restricted to soft tissue (Cases 2 and 3). The intra-osseous lesion arose in a 7-year-old girl, whereas the gingival lesions were observed in a 12-year-old girl (Case 2) and a 13-year-old boy (Case 3). Histopathologically, all cases were composed of spindle shaped cells arranged into long fascicles showing mild to moderate degree of nuclear atypia. Ki-67 (MIB-1) proliferation activity was relatively low, amounting to 3–5% in all cases. Immunohistochemically, all cases showed smooth muscle actin (SMA) positivity in spindle cells, while desmin, beta catenin, cytokeratin, and CD34 were negative, resulting in a diagnosis of LGMS. In conclusion, current series of three cases of LGMSs that occurred in the oral cavity in a child and two adolescent patients is presented to highlight an emerging disease that requires additional data for further characterization.

## 1. Introduction

Myofibroblasts are cells that share features of both fibroblasts and smooth muscle cells but differ from both cells due the presence of characteristic fibronexus and stress fibers that are identified ultra-structurally [1,2,3]. Myofibroblasts are a component of (1) granulation tissue that occurs in repair phenomena, (2) pseudosarcomatous proliferations, (3) stromal response to neoplasia, and (4) benign and malignant neoplasms of myofibroblast origin [2].

Low-grade myofibroblastic sarcoma (LGMS), which is a malignancy of myofibroblast cells, came into existence as a distinct entity following the establishment of diagnostic criteria by Mentzel et al. in 2001 [3]. It is an unencapsulated tumor composed of spindle cells arranged into fascicles with or without stellate-shaped cells showing infiltrative margins. The spindle cells have elongated and tapered nuclei showing mild to moderate degree of nuclear atypia with variable amounts of pale pink to amphophilic cytoplasms [1,2,3]. LGMSs show variable immunophenotypes, namely, smooth muscle actin (SMA)+/desmin−, SMA−/desmin+, and SMA+/desmin+, together with most cases showing positive reaction for fibronectin and calponin [3].

Although this malignancy contains the words “low grade” in its name, approximately one-third of the lesions that occur in the head and neck may behave as “high-grade” malignancies showing increased recurrences and metastasis [4]. According to Cai et al. [4], myofibroblastic sarcomas with >6 mitosis per 10 High power fields (HPF) and/or with spontaneous necrosis were shown to behave more aggressively, accompanied by a high mortality rate. In contrast, Chan et al. [5] indicated only older age as being significantly associated with poor survival.

According to literature [4,5,6,7,8,9,10,11], the majority of LGMSs have occurred in adult patients, although there is a wide age distribution from infants to elderly. Similarly, there is controversy regarding the site predilection, as well as the fact that the majority of the reports show predilection to head and neck sites including the oral cavity [3,4,6], in contrast to a few reports that indicate extremities and trunk as the site of predilection [5,8]. Within the oral cavity, the lesions may occur within the jaw bones or in the soft tissues of the tongue, gingiva, or buccal mucosa [3,4,9,10,11].

The aim of this report was to present three new cases of LGMS that occurred within the oral cavity in children. In addition, its diagnostic challenges, differential diagnoses, and prognostic aspects are discussed with a literature review-based comparison.

## 2. Case Reports

### 2.1. Case 1

A 7-year-old female patient was presented to the Dental Hospital, Peradeniya, with the complaint of a rapidly enlarging lesion in the lower jaw of three weeks duration. According to the parents, the lesion became visible following the extraction of a tooth in the vicinity. On examination, the swelling was observed both extra-orally and intra-orally, with a 3 × 4 cm swelling observed in relation to the gingiva of the lower left molar region. The first permanent molar was partially erupted and visible in the oral cavity. The lesion was pink in color, irregular in shape, and firm in consistency. Orthopantomogram (OPG) revealed an intra-osseous radiolucency that extended from first primary molar to first permanent molar region on the left side of the mandible. The developing tooth bud of the second premolar tooth was present within the lesion.

**Figure 1:**
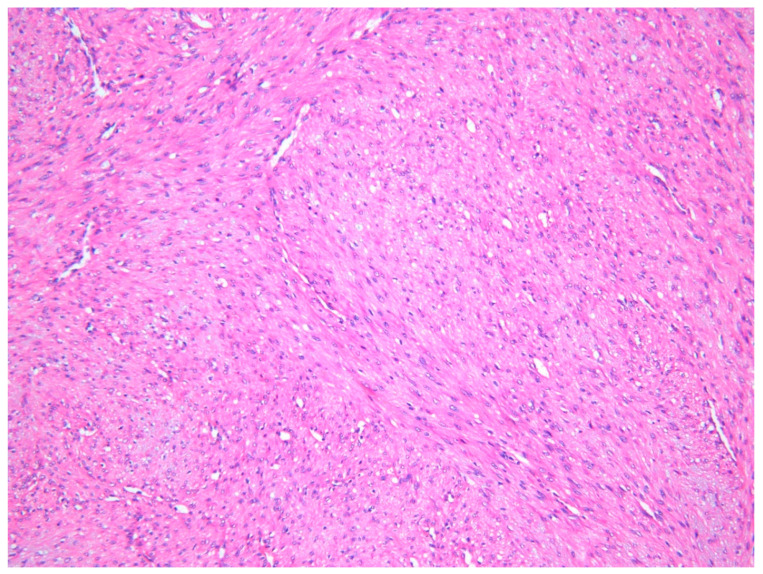
Photomicrograph exhibiting low-grade myofibroblastic sarcoma (LGMS) composed of spindle-shaped cells arranged into fascicles—Case 1 (hematoxylin and eosin stain ×10).

**Figure 2:**
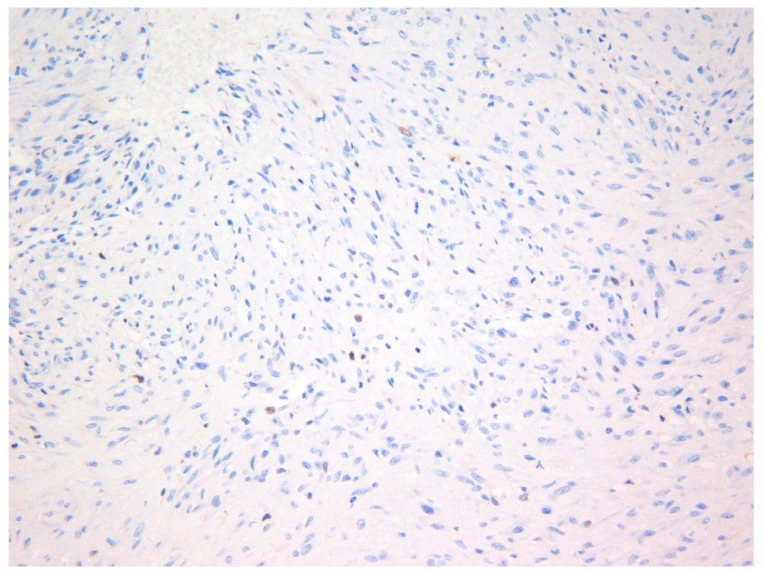
Photomicrograph showing Ki-67 (MIB-1) positivity in tumor cells—Case 1 (×40).

**Figure 3:**
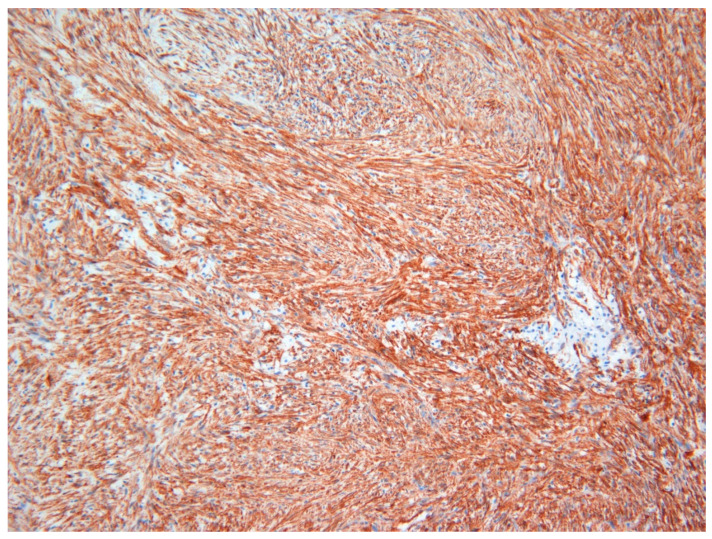
Immunohistochemical investigations with smooth muscle actin (SMA) revealed strong positivity in tumor cells in Case 1 (×20).

The lesion was excised under general anesthesia using an intraoral approach, with the specimen consisting of two soft tissues masses together measuring 3.0 × 2.5 × 1.5 cm in size with soft and hard tissue removed from buccal and posterior margins, respectively. The hematoxylin and eosin-stained section revealed an un-encapsulated tumor composed of spindle cells arranged into long fascicles (Figure 1). The tumor cells showed vesicular nuclei and eosinophilic cytoplasms. The cells exhibited a mild degree of cytological atypia with moderately high mitotic counts (8 per 10 HPF). In contrast, Ki-67 activity was 5 per 100 cells (Figure 2). Stag horn-like hemangiopericytomatous vasculature was evident throughout the lesion. These histopathological findings together with the immunohistochemical findings of smooth muscle actin (SMA) (Figure 3) and vimentin positivity (Table 1) were consistent with the diagnosis of low-grade myofibroblastic sarcoma. As the surgical margins were positive for tumor, re-excision was performed to obtain clear margins. The patient remained free of disease 12 months after the re-excision.

### 2.2. Case 2

A 12-year-old female patient presented with a recurrent gingival swelling of one-month duration. A gingival lesion that had occurred in the same region (between right upper first and second premolar) three months earlier had been excised and diagnosed as a fibrous epulis at a different center. On examination, a 5 × 2 cm swelling that involved both buccal and palatal gingiva was observed. The ulcerated lesion was pink in color, semi-hard in consistency, and oval in shape. Radiological investigations revealed superficial bone erosion only. The patient underwent right side posterior partial maxillectomy under general anesthesia using an intra-oral approach. The resection included the tumor and surrounding soft tissue and bone in order to achieve a clear margin sparing the noninvolved anterior maxilla.

The specimen consisted of two soft tissues masses and a few bone fragments, together measuring 4.5 × 5.0 × 4.0 cm in size. The hematoxylin and eosin-stained section revealed an ulcerated and un-encapsulated tumor composed of spindle cells arranged into fascicles. The tumor cells showed vesicular nuclei and eosinophilic cytoplasms. The cells exhibited moderately high mitotic counts (6 per 10 HPF) mainly in areas underneath ulceration. Prominent foci of necrosis were observed. These histopathological findings together with the immunohistochemical findings (Table 1) were consistent with the diagnosis of low-grade myofibroblastic sarcoma. Further, investigations did not reveal metastatic tumors at other sites. The patient remained tumor-free for the 15-month follow-up period.

### 2.3. Case 3

A 13-year-old male presented with a gingival swelling in the posterior mandible of one-month duration. On examination, a 4 × 2 cm pedunculated swelling that involved both buccal and lingual gingiva was observed, although the buccal extension was more compared to lingual extension. The lesion was reddish in color, oval in shape, and firm in consistency. The second molar tooth was mobile. Radiological investigations revealed bone resorption in relation to the first and second molar teeth. The lesion was conservatively removed with a soft tissue margin of normal tissue under general anesthesia.

**Figure 4:**
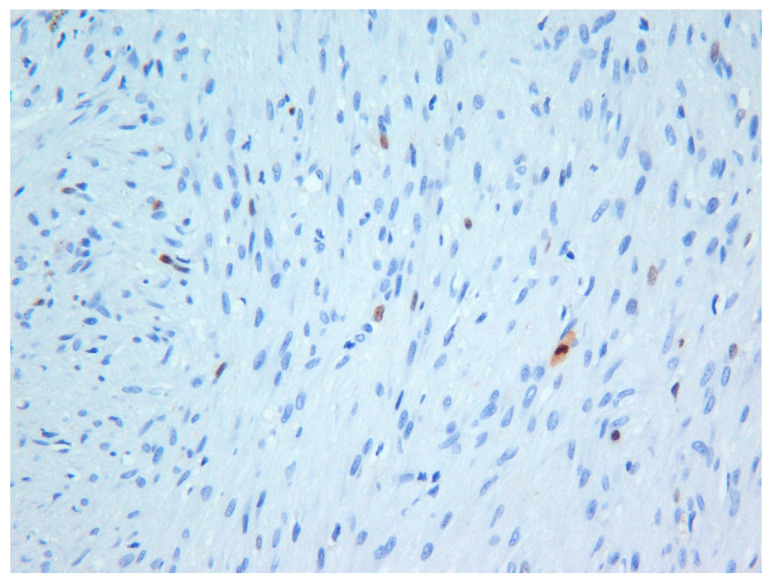
Photomicrograph exhibiting low Ki-67 (MIB-1) activity in the tumor—Case 3 (×20).

The excisional biopsy consisted of one large soft tissue mass measuring 3.0 × 2.0 × 2.0 cm in size. The hematoxylin and eosin-stained sections revealed an un-encapsulated tumor composed of spindle cells arranged into interlacing fascicles. Although the tumor cells were morphologically bland, the cells exhibited focal moderately high mitotic counts (6 per 10 HPF). However, Ki-67 (MIB-1) activity was approximately 2% (Figure 4). The histopathological findings together with the immunohistochemical findings (Table 1) were consistent with the diagnosis of low-grade myofibroblastic sarcoma. Unfortunately, the patient was lost to follow-up.

Immunohistochemical findings of all three cases are summarized in Table 1. All three patients and their guardians at the time of taking biopsies for diagnostic purposes gave consent for publishing material with de-identification as per Standard Operating Practices of Ethics Review Committee of Faculty of Dental Sciences, University of Peradeniya, Sri Lanka.

## 3. Discussion

LGMS is a rare and recently described tumor that requires further characterization because, although the name itself implies that the behavior of the malignancy is that of a “low-grade tumor”, some myofibroblastic sarcomas have presented with high grade features and behavior as well [4]. Further, due to its rarity, with less than 150 cases reported in the English language literature, it is difficult to arrive at conclusions regarding the outcome [3,4,5,6,7,8,9,10,11]. Published work on LGMS reveals a relatively high percentage of tumors to have occurred in the oral cavity [4,6,7,8,9,10,11]. However, all reports published to date have analyzed LGMS of the head and neck region that included the tumors of the oral cavity, making it difficult to establish features of oral LGMS [4,9,10,11]. Therefore, we analyzed the clinical presentations of LGMS that occurred purely in the oral cavity on the basis of cases reported in the literature (Table 1 and Table 2). Altogether, 33 LGMSs were included in the analysis (30 cases from the literature and the 3 new cases from the present report).

Accordingly, there were 13 cases of LGMS that occurred in the jaw bones, while the rest (20 cases) were involved purely soft tissues of the oral mucosa. When we considered both intra-osseous and extra-osseous lesions, the lesions occurred in a wide age range from 7 to 74 years, with a slight male predilection (male to female ratio 1:0.7) (Table 3). With reference to the intra osseous lesions, the majority occurred in the posterior region of the jaw bones. A bimodal age distribution could be observed, with reference to mandibular lesions, namely, at one extreme the lesions occurring in children and adolescents whist at the other extreme the lesions occurring in elderly patients in their sixth to eighth decades of life (Table 2). In contrast, when LGMS of oral soft tissues were considered, the lesions seemed to be more common in adult patients (Table 2 and Table 3), showing a similar age distribution to LGMS of other sites [3,4].

It is also very difficult to predict the outcome of LGMS, as a majority of the published cases have follow-up information for very short periods of time, i.e., the majority were followed up for less than 36 months (Table 2). The lack of long-term follow-up is a major limitation of the cases included in the present study as well. Therefore, new reports with longer follow-up periods are a “need of the hour”. Although the LGMSs have been followed up for short periods of time, approximately 30% of lesions have presented with recurrences (Table 3) [4,10]. Further, when intra-osseous lesions were considered, LGMS of maxilla showed a very high recurrence rate (80%) compared to mandibular lesions (Table 3) [4,10]. Two reasons can be proposed for the high recurrence rate of maxillary lesions, namely, the potential relative difficulty to obtain complete surgical clearance due to proximity of vital structures and/or due to the fact that maxillary bone is relatively thin compared to mandibular bone, allowing rapid spread of the tumor. In addition, type of surgical treatment (whether conservative or radical) received could also contribute to the high rate of recurrence observed in maxillary lesions. Although it would have been worthwhile to correlate the surgical approach taken with the recurrence rate, it was not possible, as most reports available in the literature do not describe the surgical approach in detail.

LGMS is a diagnostically challenging tumor that requires immunohistochemical (Table 1) and/or electron microscopical investigations to establish a definitive diagnosis [1]. Even with the immunohistochemical investigations, it is a difficult diagnosis, as myofibroblsts do not have specific immunohistochemical markers for its identification. Smooth muscle actin that is used for the identification of myofibroblasts can be used to identify tumors of smooth muscle origin, while desmin can be used to identify tumors of skeletal muscle origin as well [1,4].

The differential diagnoses of LGMS include myofibroma, spindle cell carcinoma, nodular fasciitis, desmoids-type fibromatosis, inflammatory myofibroblastic tumor (IMT), solitary fibrous tumor, hemangiopericytoma, leiomyosarcoma, juvenile variant (spindle cell variant) of rhabdomyosarcoma, and fibrosarcoma [1]. Myofibroma was considered in the differential diagnosis of Case 1 as the lesion showed numerous stag horn-shaped blood vessels (hemagiopericytomatous vasculature). However, the lesion did not show characteristic biphasic arrangement of cellular areas with closely packed cells and sparsely cellular areas containing spindle cells, which is characteristic of myofibroma [21], and instead showed spindle cells arranged into long fascicles. Further high mitotic activity was also useful to exclude the myofibroma in the patient.

When LGMS of oral cavity was considered, nodular fasciitis could be excluded as it occurs at extra oral sites. Further, Meng et al. have shown that the two entities are different genetically, with LGMS showing complex DNA copy number changes that are not observed in nodular fasciitis [22]. IMT can be distinguished from LGMS by the fact that former lesion express ALK (anaplastic lymphoma kinase) and cytokeratin [4]. Solitary fibrous tumor/hemangiopericytoma (CD34, BCL2, and CD99 positive), leiomyosarcoma (SMA and H-CALDESMON positive), and juvenile variant (spindle cell variant) of rhabdomyosarcoma (desmin and MYO-D positive) can be differentiated from LGMS by immunophenotypical differences (Table 1) [1].

Cai et al. [4] indicates >6 mitotic figures per 10 HPF and spontaneous necrosis as features of high-grade behavior. All three new cases included showed high-grade features, namely, >6 mitotic figures, while Case 2 showed spontaneous necrosis. However, in contrast to mitosis, Ki-67 (MIB-1) activity was generally rather low in all three cases. Presence of ulceration (Case 2) may result in higher mitotic counts when compared to Ki-67 (MIB-1) activity. Technical aspects such as problems during tissue fixation may be another reason for the low Ki-67 proliferation rate observed in the present cases. As such, the dilemma of whether to use mitotic activity or Ki-67 (MIB-1) proliferation rate to base conclusions on “high-grade behavior” should be resolved with further evidence. Furthermore, although we diagnosed the lesions as “low-grade” myofibroblastic sarcomas, close follow-up is mandatory for these cases as they have the “high-grade” features mentioned above.

Surgical resection with the aim of achieving clear margins is the current gold standard management for LGMS [9,23]. Xu et al. [23], on the basis of follow-up data on 96 patients, concluded that surgical resection is the most effective therapy for LGMS. In addition, they mention that chemotherapy and/or radiation therapy should not be routinely performed in LGMS, especially for those with negative margins after surgery.

In conclusion, according to the clinicopathological features, LGMS of oral mucosa may occur in both children and adults. Recurrences can be commonly observed in maxillary lesions. However, long-term follow-up information is required before definite conclusions can be made regarding the outcome.

## Figures and Tables

**Table 1:** Immunohistochemical investigations performed for Cases 1–3.

Case Number	Immunohistochemical Findings	
	Positive	Negative
Case 1	SMA	S-100
	Vimentin	Cytokeratin
		Desmin
		CD99
		Β catenin
Case 2	SMA	S-100
		Cytokeratin
		Desmin
		CD9
Case 3	SMA	S-100
	Vimentin	Cytokeratin
		Desmin
		CD99

**Table 2:** Clinical features of low-grade myofibroblastic sarcomas of the oral cavity obtained from the literature and the three new cases [4,6,7,9,10,11,12,13,14,15,16,17,18,19,20].

Reference (Number & Citation)	Age	Gender	Site	Outcome
**Intra-Osseous Lesions**				
[4] Cai (2013)	74	F	Mandible	NED at 4 months
[9] Demarosi (2009)	51	M	Mandible	NED at 12 months
[10] Qui (2014)	45	M	Maxilla	NED at 30 months
	29	F	Maxilla	Recurrence at 6 months
[13] Smith (1995)	12	F	Maxilla	Recurrence at 9 months
	18	M	Maxilla	Recurrence at 24 months
	9	F	Mandible	Recurrence and metastasis at 8 months
	9	F	Mandible	NED at 96 months
[14] Mentzel (1998)	19	M	Mandible	NED
[15] Bisceglia (1999)	49	M	Maxilla	DOD at 36 months
[16] Keller (2004)	8	F	Mandible	NED at 72 months
Current Case 1	7	F	Mandible	NED at 12 months
**Extra-Osseous Lesions**				
[4] Cai (2013)	17	M	Lip	NED at 12 months
	44	M	Tongue	NED at 02 months
	14	M	Palate	N/A
[6] Montgomery (2001)	41	M	Hard palate	NED at 24 months
	35	M	Palate	Recurrence present
	54	F	Gingiva	Recurrence present
[7] Meng (2007)	53	M	Tongue	Recurrence at 24 months
[9] Demarosi (2009)	61	F	Tongue	NED at 24 months
[11] Montebugnoli (2010)	37	M	Gingiva	NED at 18 months
[12] Eyden (1992)	43	F	Buccal mucosa	NED at 24 months
[14] Mentzel (1999)	51	M	Tongue	NED
	70	M	Tongue	NED
	24	M	Tongue	NED
	66	M	Tongue	NED
[17] Artopoulou (2006)	37	F	Buccal mucosa	NED at 6 months
[18] Laco (2006)	24	F	Tongue	NED at 12 months
[19] Jay (2009)	41	M	Tongue	Recurrence present, NED at 36 months
[20] Yamada (2012)	73	M	Palate	NED at 24 months
Current Case 2	12	F	Gingiva	Recurrence present, NED at 15 months
Current Case 3	13	M	Gingiva	N/A

NED—no evidence of disease, DOD—died of disease, N/A—not available, F—female, M—male.

**Table 3:** Literature review-based comparison of clinical presentations of low-grade myofibroblastic sarcoma depending on the site of occurrence.

Location	Age—Mean (Range)	Gender	Status of Recurrence
**Intra-Osseous Presentation**			
Maxilla (*n* = 5)	30.6 years (12–49 years)	F = 2, M = 3	Present = 4
Mandible (*n* = 8)	35.2 years (7–74 years)	F = 5, M = 3	Present = 1
**Extra-Osseous Presentation**			
Tongue (*n* = 9)	48.2 years (24–70 years)	F = 3, M = 6	Present = 2
Palate (*n* = 4)	40.7 years (14–73 years)	F = 0, M = 4	Present = 1
Gingiva (*n* = 4)	29.0 years (12–54 years)	F = 2, M = 2	Present = 2
Other (*n* = 3)	32.3 years (17–43 years)	F = 2, M = 1	Present = 0
**Total**	36 years (07–74 years)	F = 14, M = 19	Present = 10
